# Inhalation Exposures to Particulate Matter and Carbon Monoxide during Ethiopian Coffee Ceremonies in Addis Ababa: A Pilot Study

**DOI:** 10.1155/2010/213960

**Published:** 2010-09-21

**Authors:** Chris Keil, Hailu Kassa, Alexander Brown, Abera Kumie, Worku Tefera

**Affiliations:** ^1^Department of the Environment and Sustainability, Bowling Green State University, Bowling Green, OH 43403, USA; ^2^Department of Public and Allied Health, Bowling Green State University, Bowling Green, OH 43403, USA; ^3^School of Public Health, College of Health Sciences, Addis Ababa University, Addis Ababa, Ethiopia

## Abstract

The unique Ethiopian cultural tradition of the coffee ceremony increases inhalation exposures to combustion byproducts. This pilot study evaluated exposures to particulate matter and carbon monoxide in ten Addis Ababa homes during coffee ceremonies. For coffee preparers the geometric mean (57 *μ*g/m^3^) and median (72 *μ*g/m^3^) contributions to an increase in a 24-hour time-weighted average exposure were above World Health Organization (WHO) guidelines. At 40% of the study sites the contribution to the 24-hour average exposure was greater than twice the WHO guideline. Similar exposure increases existed for ceremony participants. Particulate matter concentrations may be related to the use of incense during the ceremony. In nearly all homes the WHO guideline for a 60-minute exposure to carbon monoxide was exceeded. Finding control measures to reduce these exposures will be challenging due to the deeply engrained nature of this cultural practice and the lack of availability of alternative fuels.

## 1. Introduction

Cooking with biomass, especially inside structures with poor ventilation, creates significant health hazards. The World Health Organization (WHO) [1] estimates that there are 1.5 million deaths per year because of exposure to indoor air pollution and that a large proportion of those affected are women and children. More than one half of the world's population relies on biomass for energy needs. Biomass fuels, including wood, dung, agricultural residues and charcoal, are large contributors to indoor air pollution levels. Combustion of these fuels results in emissions of carbon monoxide (CO), nitrogen dioxide (NO_2_), a variety of organic vapors, and particulate matter that contain a complex mixture of heavy organic compounds. As summarized by Smith [2], health effects with evidence of a strong association with indoor air pollution from biomass combustion include acute respiratory infection, chronic obstructive pulmonary disease, lung cancer, tuberculosis, cataracts, asthma attacks, and adverse pregnancy outcomes. 

Studies specific to East Africa demonstrate that air pollutants originating from biomass are indeed problematic in that region. Kumie et al. [3] found that in one region in Ethiopia the level of NO_2_ in households that used biomass fuel for cooking was twice the WHO guideline for NO_2_ concentrations. A study in rural southwestern Ethiopian communities [4] documented the biomass fuel-related problems. These included the conditions that produced exposure such as no separate kitchen and lack of windows and elevated particulate matter concentrations. Another study in rural northern Ethiopia documented that 80% of cooking was done indoors with biomass [5]. In this same study only 13% of the women thought the smoke exposures were of concern. In nearby Kenya, Ezzati and Kammen [6] studied the effects of pollutants from biomass combustion on health in rural areas and found that acute respiratory infection increased with increasing exposure to particulates with a mass median aerodynamic diameter less than 10 *μ*m. This same study indicated that due to gender roles (including cooking), females spend more time indoors than outdoors, and thus they may have higher exposures to indoor air pollution than adult males. The exposures are likely to be higher for females in rural areas, where the “3-stone fire” is traditionally used for cooking compared to urban and semiurban, where ceramic wood stoves or charcoal stoves are more likely to be used for cooking. 

In the Ethiopian context, exposures to air pollutants from biomass combustion may be further elevated due to the unique cultural tradition of the “Coffee Ceremony”. The coffee drinking ritual plays a major role in the spiritual and cultural life of the people and is an excellent example of the hospitality of Ethiopians towards visitors. Coffee is brewed and offered to guests as a sign of friendship and respect, during celebration or mourning as well as generally in the morning with breakfast before going to work and occasionally in the afternoon after lunch and during evening hours with dinner. The processes of the ceremony, however, result in the release of air pollutants.

The traditional coffee making preparation involves several steps and is generally brewed by a young woman in the household. The entire process is typically performed indoors in the room where the participants are seated. The coffee beans are first washed and then roasted on a flat pan over a small charcoal stove. In rural areas coffee is roasted mainly over “3-stone fire” using biomass fuel such as cow-dung, crop residues, wood, or charcoal. The coffee is roasted by turning it over and over again using a small metal or wooden stick bent in at one end for easy turning of the coffee beans. This is done until the beans turn black and shinny and the aromatic oil is coaxed out of the coffee. Once the beans are roasted, the preparer takes the coffee pan around the room to each guest shaking the pan occasionally until the room is filled with the coffee aroma. The guests may pull the coffee aroma towards themselves by moving their hand back and forth towards their face. During the ceremony, from start to finish, incense is often burned in a small burner made from clay. There are two types of incense that may be used, ital and sendel. 

After roasting, the coffee beans are poured into a heavy wooden bowl and crushed and ground manually with a wooden or metal stick in up and down motion, like a mortar and pestle. The ground coffee is then put into a small pot to which water is added. The pot is then put over the charcoal stove or fire until the mixture boils. The boiled coffee is allowed to settle for a minute to separate the liquid from the coffee grounds. The coffee is served in a small ceramic cup. The cups are arranged in a coffee tray close to each other, and then the preparer fills each cup with the coffee and gives a cup to each guest. Traditionally, coffee is served in three rounds by adding water in second and third round to the coffee sediment which is then brought to a boil again and served as described above. The third serving is considered to bestow a blessing and the ceremony is traditionally closed by an elderly person saying a prayer.

Although the coffee ceremony is an important cultural and traditional ceremony bringing people together to discuss personal and societal issues, the combustion byproducts emanating from the coffee preparation and incense may pose health risks to people exposed to the smoke. 

The question to be addressed in this study is whether exposures to combustion byproducts during the coffee ceremony pose increased health risks to the coffee preparer and participants in the ceremony. While there are many combustion byproducts, this study measured particulate matter and carbon monoxide (CO) concentrations. Particulate matter and CO have been identified as good indicators of health hazards from biomass smoke [2].

## 2. Methods

### 2.1. Subject Recruitment

Ten homes in Addis Ababa were selected for participation in this pilot study. The selection was nonrandom and was drawn from a network of individuals known to the authors. Specific homes were selected in an effort to evaluate a variety of types of homes and building construction in Addis Ababa. 

The research team attended one full coffee ceremony at each home. All homes used a charcoal brazier to prepare the coffee. 

Informed consent was obtained from all the coffee preparers. A written informed consent form was developed and translated into Amharic. The consent procedures were approved by human subject review boards at both Bowling Green State University and Addis Ababa University.

### 2.2. Data Collection

Prior to the coffee ceremony each coffee preparer was orally asked questions regarding her attitude toward the smoke created during the ceremony and her preference of heating methods. The questions, to be detailed later, were open ended and the preparer was free to respond orally. The coffee preparer was reimbursed 100 birr (equivalent to about US $8) for her time as well as the cost of the materials for the ceremony (coffee beans, fuel, incense). 

At each home the research team measured the dimensions of the room in which the ceremony took place and made qualitative notes of the airflow in the room and degree of natural ventilation. An ordinal scoring system was used to describe the availability of natural ventilation. The number of open doors and windows in the room was recorded. If a door or window opened directly to the outside it was scored as a 1. A score of 0.5 was used for each open door that led to a room that was in turn open to the outdoors. These scores were summed to determine a “natural ventilation” score for the room that represented the relative amount of natural ventilation available in the room. None of homes had mechanical ventilation. 

Carbon monoxide was measured using a Lascar EL-USB-CO carbon monoxide monitor. This monitor consists of an electrochemical sensor and data logging circuitry. The sensor operates by CO molecules entering a detection cell via a capillary. The CO interacts with an electrode undergoing oxidation to carbon dioxide. The electrons liberated by the reaction produces a measurement current which is logged. The sensor meets the requirements of all international performance standards. The monitor is built with a USB connection for direct connection to a computer and download of the logged data using a manufacturer provided software interface. The data can be exported to formats for commonly available data analysis software. The factory calibration of the instrument was confirmed with a zero-span calibration check before departure for Ethiopia and again upon return to the United States. 

**Figure 1:**
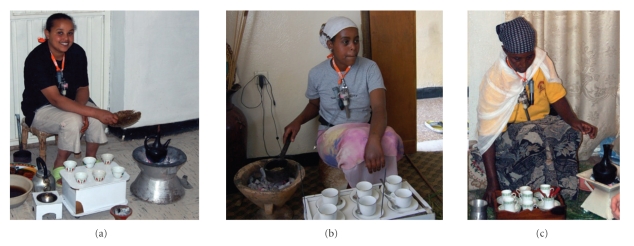
Personal sampling on preparers.

A personal CO sample was taken on the preparer. An area sample was taken in the vicinity of the other participants in the ceremony, one to two meters from the preparer. The preparer wore the monitor on a lanyard around her neck. Figure 1 illustrates the personal sampling setup.

Particulate matter was measured using National Institute for Occupational Safety and Health Methods 0500 and 0600 for total particulate matter and respirable particulate matter [7]. Respirable particulate matter is defined by the International Organization for Standardization and the European Standardization Committee as having a mass median aerodynamic diameter of 4.0 microns (PM_4_) [8, 9]. Preweighed PVC filters were used for both total and respirable sampling. Respirable samples had a 10 mm nylon cyclone inlet to separate the respirable from total particulate matter. Personal sampling pumps were used to pull air through the sampling trains. Sampling train flow rates were calibrated immediately before sampling and checked immediately after sampling. The mass gain on the filter was determined by an American Industrial Hygiene Association accredited laboratory. 

A respirable particulate matter sample was taken on the preparer. She wore the sampling pump on a belt and the cyclone/filter assembly was worn around on the same lanyard holding the CO monitor. Side by side total and respirable particulate matter samples were also taken near the participants in the ceremony, one to two meters from the preparation of the coffee. Sampling began when coffee bean preparation began. Sampling ended at the pouring of the third cup of coffee.

### 2.3. Statistical Methods

When particulate matter sample results were reported by the laboratory as below the limit of detection (LOD), the values were treated as having a value of LOD × 1/2^0.5^ for data analysis and statistical tests [10]. 

A Shapiro-Wilks *W*-test determined that the concentration data was better described as log-normally distributed than normally distributed. Because of this the geometric mean (GM) and geometric standard deviation (GSD) were used as descriptors of central tendency and variability. ANOVA analysis was done to determine if the factors: type of construction, room geometry, ventilation descriptions, and incense use had an effect on pollutant levels. Log transformed concentrations were used for the ANOVA test since the data was log-normally distributed.

The average concentration during the coffee ceremony and the length of time each day that the coffee ceremony took place were used to determine the contribution to the 24-hour time weighted average respirable particulate matter exposures from the coffee ceremony. These were compared to the WHO 24-hour mean concentration guideline for PM_10 _ which is 50 *μ*g/m^3^ [11]. 

## 3. Results and Discussion

### 3.1. Sampling Site and Ceremony Information

The homes visited represented the spectrum of housing types in Addis Ababa. The ability to arrange access prevented the homes visited to be representative of the prevalence of these types in the city. Two homes were mud houses with dirt floors. This type is typical of the poor class and by far the most common type of home in the outskirt of Addis Ababa. Four homes were wooden frames covered with cement/plaster and poured concrete floors. This type of home is typical of working class families. Four homes were brick or cinderblock covered by plaster, typical of the professional class and the upper class. For the most part, different types of homes could be found within a single block of a neighborhood. Next to a modern house of an upper class family, it is not uncommon to see a mud house of lower or poor class family. 

The homes visited varied in terms of how often the coffee ceremony was done. In one house the preparer reported holding the coffee ceremony once every two or three days. In another home the frequency was three times a day. The median ceremony frequency was twice a day. The duration of the ceremony ranged from 60 to 85 minutes with a median of 71 minutes. The median volume of the room that the ceremony took place in was 44 m^3^ with a range from 22 m^3^ to 101 m^3^. The preparers were all female ranging in age from 15 to 50. Additional individuals often participated in the ceremony along with the members of the research team. Table 1 provides descriptive information on the sampling sites.

The coffee preparers' responses regarding their concern about their inhalation exposures during the ceremony are presented in Table 2. Half of the preparers expressed concern over their exposures.

### 3.2. Air Pollutant Concentrations

Tables 3 and 4 present the results of the air sampling. The geometric mean of particulate matter concentrations was over 1000 *μ*g/m^3^. Carbon monoxide concentrations exceeded the WHO guidelines [12] at a number of the sites. The one-hour guideline was exceeded in at least one sample in nine of the ten households.

### 3.3. Factors Affecting Concentrations

The effect of the type of construction, room geometry, ventilation descriptions, and incense use on pollutant levels were evaluated using ANOVA analysis. The only significant difference was that average concentrations of personal respirable particulate matter were lower in the homes that did not use incense. This must be interpreted with caution since the respirable concentrations in the houses that did not use incense were below the limit of detection and statistical estimates of concentrations were used. 

At eight sites incense was used and at least one of the respirable particulate matter samples was above the LOD at each of those sites. This compares with no particulate matter samples above the LOD at either of the two sites where incense was not used. Unfortunately the type of incense used was not consistently recorded. The effect of the different types of incense will be the subject of future work. 

At half of the sites using incense the concentration measured by the area sample was higher than the preparer's personal sample. This is interesting since the preparer was seated quite close to the incense burner. The visible plume of particulate matter from the incense typically rose in a laminar stream to a height above the breathing zone of the preparer. This may account for the higher concentrations in the area samples than the personal samples, but more work is required to draw conclusions.

Though the particulate matter concentrations appeared to be lower in homes that did not use incense, the practice of using incense did not seem to impact CO levels. In fact one of the homes that did not use incense had the second highest CO levels. A tentative explanation that needs further investigation is that incense is the more significant contributor to particulate matter emissions and the charcoal is the primary contributor to CO levels. A third source of particulate matter, the coffee bean roasting process, cannot be addressed independently based on the current data.

### 3.4. Health Impacts

Particulate matter has a complex chemical makeup that varies widely over time at a given location as well as from location to location. There may be specific health effects resulting from the toxicology of a specific chemical component of particulate matter. For example lead is a USEPA criteria pollutant and is regulated on a chemical concentration basis without regard to the size fraction. Independent of the chemical composition, the size of particulate matter is directly related to their potential to cause health problems. The health impact of particulate matter is assessed by measuring the concentration of particulate matter in a given size range, since even the most chemically less toxic particulate matter can produce a health effect if it is small enough to be deposited deep in the lungs. 

In this pilot study respirable particulate matter exposures were measured because of the well-established convention and methodology used to assess risks from occupational exposures to particulate matter. The Threshold Limit Value (TLV) for respirable particulate matter is 3,000 *μ*g/m^3^ as an 8-hour time weighted average [13]. However, occupational exposure limits for respirable particulate matter should not be used to evaluate risks due to community exposure [13]. Occupational exposure limits such as the TLV are not fine lines of safety, but are developed to protect most workers. They also assume that the worker population is a generally healthy worker population with a small proportion of young, old and other susceptible populations. For these reasons, occupational exposure limits for respirable particulate matter are of limited value in assessing the risks from the exposures related to the coffee ceremony.

Respirable particulate matter represents the size of particulate matter likely to penetrate to the gas exchange regions of the lungs. This size fraction has an aerodynamic diameter cut point of 4 *μ*m. In the ambient/community air pollution context the thoracic size fraction is often evaluated. The thoracic fraction is the particulate matter size fraction that will penetrate the head region, enter the thoracic region of the lungs, and may penetrate to the gas exchange region. This thoracic fraction has a cut point of 10 *μ*m and is referred to as PM_10_. Thus respirable particulate matter is a subfraction of PM_10_. By measuring respirable particulate matter, a minimum PM_10_ concentration is being established since the PM_10_ concentration would include particulate matter 4–10 *μ*m as well as the respirable particulate matter, which is PM_4_ and smaller.

To gain some insight regarding the health risk posed by the smoke exposures from the coffee ceremony, the respirable particulate matter exposures can be used as a minimum estimate of PM_10_ exposures as described above. Epidemiologic studies have not identified a clear health effect-exposure threshold level. Studies have demonstrated an increase in mortality of approximately 0.50% per 10 *μ*g/m^3^ increment in daily concentrations [11]. The WHO provides a 24-hour mean ambient exposure guideline for PM_10_ of 50 *μ*g/m^3^ [11]. This guideline was set to protect against cardiopulmonary and lung cancer mortality.

The exposures in this study were measured for a shorter duration and cannot be directly compared to the 24-hour guideline. Though the coffee ceremony is a discrete event that does not continuously contribute to particulate exposures, the duration and frequency of the ceremony do contribute to an increase in an individual's long-term particulate exposure. For each location the contribution to the 24-hour average particulate matter exposure was calculated as follows.

- Fraction of day at ceremony = [Duration (min) of an ceremony × Average ceremonies per day]/1440 (min) per day.
- Contribution to 24-hour exposure = (i) × Exposure at the ceremony.

For example, at site 1 the respirable particulate matter exposure of the cook was 2100 *μ*g/m^3^ and the ceremony was performed on average 3 times a day with a length of 82 minutes. So at that site a total of 246 minutes a day or 17% of the day was spent at the coffee ceremony with a respirable particulate matter concentration of 2100 *μ*g/m^3^. If this was the only respirable particulate matter exposure during the day, the 24-hour average exposure would be 360 *μ*g/m^3^. Therefore on average the coffee ceremony contributed 360 *μ*g/m^3^ to the 24-hour exposure of the cook at this site. Table 5 presents the coffee ceremony related increase in 24-hour exposures for cooks and participants. 

At seven of the ten sites, the 24-hour average particulate matter exposures from the coffee ceremony alone, apart from other ambient exposures, are above the 2005 WHO guideline. The mean and median concentration increases are both above the same guideline. This suggests that the coffee ceremony could be a contributor to the frequency of respiratory illness related deaths.

These analyses are limited by a number of factors. Firstly, many of the health effects studies were made in the North American or European context. While there is no *a priori* reason to assume that physiologic responses among East Africans would be markedly different, the studies used to determine the relative risks were done in developed countries. Secondly the exposure response curves are described as fitting a straight line “reasonably well” at low levels (0–100 *μ*g/m^3^). The short-term exposures measured in this project were much higher than that. For 40% of the locations even the time-weighted 24-hour average increases in particulate matter exposure were greater than 100 *μ*g/m^3^. Some studies have shown that at concentrations of several hundreds of *μ*g/m^3^ the slope of the response becomes shallower [12]. Thirdly, respirable particulate matter concentrations were measured. Actual PM_10 _ exposures would have been higher as was described above. 

Regarding carbon monoxide exposures, the WHO CO guidelines are set to protect individuals with coronary artery disease from acute ischemic heart attacks and to protect fetuses from hypoxic effects [12]. The guideline for 15-minutes exposures is 90 ppm, for 30 minutes is 50 ppm, and for 60 minutes is 25 ppm [12].

In only two households were the short-term (15-minute) exposures were observed to be over the guidelines. However, the degree to which the guidelines were exceeded increased the longer averaging time. Looking at the one-hour average CO concentrations, only one location did not have at least one sample over the recommended level. At three sites the one-hour personal exposures were greater than twice the guideline. Five more of the one-hour personal samples were between 100% and 200% of the guideline. Six of the ten sites had area one-hour average concentrations greater than 25 ppm.

Coffee preparers are often young women who may at some point be pregnant. Considering that one health concern from the CO exposure is fetal hypoxic effects these exposure results may be cause for concern. The potential outcomes from fetal hypoxia are manifold. Depending on the developmental stage of the fetus when hypoxia occurs, the impacts on individuals and in the community as a whole may be significant.

The prevalence of coronary artery disease in Ethiopia is unknown. Given the typical Ethiopian diet the prevalence of coronary artery disease among the young is expected to be low. The prevalence may be higher in the elderly who are often frequent participants in the coffee ceremony. The results show that the one-hour CO guideline is often exceeded and the shorter term guidelines are also exceeded, though less often. This represents an increased risk of heart attacks in the susceptible population.

### 3.5. Degree of Concern and Controlling Exposures

Half of the cooks expressed some degree of concern about the smoke associated with the coffee ceremony. Interestingly the cooks who had the three highest respirable particulate matter exposures expressed no concern. This level of concern is higher than what was observed in a rural area [5]. In the study of the rural area, once educated the awareness level among the women increased, but they had no access to alternative cooking methods or means of control.

Any discussion of controlling inhalation exposures during the coffee ceremony must take into consideration the deeply rooted cultural role that the coffee ceremony plays. Ethiopia is a multiethnic country, yet the coffee ceremony is practiced across ethnic divisions. Any suggestions for modifications of the ceremony must be done carefully with practical considerations in mind.

There are practices that might potentially reduce smoke exposures during the coffee ceremony. Many of these however are likely to encounter cultural resistance. One set of options is to use an alternative heating method or fuel such as electricity or kerosene. The coffee preparers were asked about their opinions regarding alternative heating methods. The responses are summarized in Table 6.

There is an interesting mix of responses showing in some preparers the willingness to try alternatives. However even among the women that expressed an interest in alternative fuels, subsequent discussions often revealed that the choice was not entirely theirs to make. Though they may be willing to change, the desires of the participants in the ceremony may tend toward keeping the traditional approach. 

Other matters of cooking practice may influence smoke exposures as well. Lighting the charcoal outside then bringing it inside when ready is one option that might be explored. However, in the one household that practiced this there did not seem to be any clear effect on concentrations. Leaving doors open to allow better natural ventilation is another option, though this has not been systematically investigated in this study beyond qualitative measures.

Since it seems that incense may be a large contributor to the particulate matter concentrations perhaps the elimination or reduction of its use would decrease particulate matter exposures. It is interesting that women in this study cited religious (Ethiopian Orthodox) reasons both for using and *not* using incense in the ceremony. This could be further investigated among the Ethiopian Orthodox clergy.

Ultimately, recommending measures to reduce exposures to smoke from the coffee ceremony is premature at this point. As an initial study, the health risk estimation is preliminary and a full characterization of the sources, determinants of exposure, and possible interventions still needs to be completed.

## 4. Conclusions

The common practice of the coffee ceremony increases particulate matter and carbon monoxide exposures for both preparers and participants. These increases exceeded the WHO exposure guidelines [11, 12] in a number of cases. This pilot study was limited to Addis Ababa. Practices such as using biomass fuel other than charcoal and three-stone heating processes were not evaluated. A more systematic and larger study of coffee ceremony exposures is warranted.

## Figures and Tables

**Table 1:** Data on site geometry and coffee ceremony.

Site	Preparer Age	Incense Used?	Other Participants	Duration (min)	Times/Week	Room Area (m^2^)	Room Volume (m^3^)	Natural Vent.
1	35	Y	1M, 1F	82	21	27	81	0.5
2	27	Y	2M	85	14	30	75	0.5
3	27	Y	1M	71	4	9	22	1.5
4	15	N	1M	69	14	9	28	1.5
5	50	Y	0	62	7	8	24	1
6	37	Y	0	71	14	11	29	1
7	37	Y	2M	60	14	14	37	1
8	16	Y	1M, 2F	64	2	33	88	1.5
9	49	N	2M, 1F	77	14	21	52	1
10	18	Y	2M	81	14	38	101	0.5

Median	31	Y*		71	14	17.5	44.5	1

*: mode, M: male, F: female.

**Table 2:** Preparers' Open Responses to Question “Are you concerned about smoke exposures during the ceremony?”.

Site	Response (translated)
1	I have no problems
2	I have no fear
3	I get headaches from the smoke which makes me worry a little
4	I am not worried about the smoke
5	The incense is fine but the charcoal is probably problematic
6	I have no worries. I never thought about it
7	Yes the smoke is a problem. I start the coals outside and then bring it in when the coals are red
8	The charcoal does not bother me. I have no worries
9	I see it as a problem. It feels suffocating
10	I do not like the smoke. It makes it hard to breathe

**Table 3:** Particulate Matter Concentration Data.

Site	Averaging	Respirable Particulate Matter		Total Particulate Matter
	Time (min)	Personal Sample (*μ*g/m^3^)	Area Sample (*μ*g/m^3^)	Area Sample (*μ*g/m^3^)
1	82	2100	2500	2400
2	85	2400	4200	4700
3	71	860	1100	930
4	69	<840	<860	<710
5	62	950	<940	1000
6	71	1900	1200	2600
7	60	1600	980	<830
8	64	<920	920	<780
9	77	<760	1000	1200
10	81	<720	<720	<620

Median	71	905	846	965
GM	72	1028	1025	1088
GSD	1.1	1.83	1.97	2.24

**Table 4:** Carbon Monoxide Concentration Data (ppm).

Site	Highest 15-minute average		Highest 30 minute average		Highest 60 minute average	
	Personal	Area	Personal	Area	Personal	Area
1	120^A^	90^A^	110^B^	88^A^	95^B^	85^B^
2	58	71	55^A^	61^A^	46^A^	48^A^
3	35	36	32	27	31^A^	22
4	58	47	49	40	46^A^	37^A^
5	58	31	26	45	32^A^	19
6	56	34	32	50^A^	50^B^	31^A^
7	52	14	13	45	39^A^	13
8	37	19	15	25	21	11
9	115^A^	74	57^A^	86	74^B^	49^A^
10	38	40	33	34	24	28^A^

Median	57	38	32.5	45	42.5	29.5
GM	57	39	35	46	41	29
GSD	1.53	1.81	1.89	1.54	1.60	1.88

^A^: Exceeds the WHO guideline

^B^: Exceeds twice the WHO guideline

WHO guidelines—concentrations not to be exceeded for averaging times, 15 minute: 90 ppm, 30 minutes: 50 ppm, 60 minutes: 25 ppm [12].

**Table 5:** Coffee ceremony related contribution to 24-hour average respirable particulate matter (PM_4_) exposure of cooks and participants.

Site	Concentration Increase (*μ*g/m^3^)	
	Cooks	Participants
1	359	427
2	283	496
3	24	31
4	57	58
5	41	29
6	187	118
7	133	58
8	8	8
9	57	107
10	57	57

Median	57	58
GM	72	72

**Table 6:** Preparers' Open Responses to Question “What are your thoughts on using alternative heating methods?”.

Site	Response (translated)
1	I prefer the quality of charcoal in preparing the coffee
2	I prefers an electric stove because of the time saved
3	If I had the chance, I would try alternative fuels but charcoal is all I know
4	I do not have much reference. I sometimes use kerosene heater (used today to initially boil water), but charcoal is cheaper. I would use gas if it were less expensive
5	I have no reference having never tried any other source, but would try one if it were available
6	I have reference, but would only use charcoal for the social aspect
7	I only use charcoal because that is the tradition
8	I use charcoal only—better taste and keeps tradition
9	I use charcoal only because of the taste, ambient heat source, and slow roasting is desired for social interaction
10	I prefers gas/electric but use charcoal because it is cheaper and I am told to
